# Possible Applications of Edge Computing in the Manufacturing Industry—Systematic Literature Review

**DOI:** 10.3390/s22072445

**Published:** 2022-03-22

**Authors:** Kacper Kubiak, Grzegorz Dec, Dorota Stadnicka

**Affiliations:** 1 Faculty of Mechanical Engineering and Aeronautics, Rzeszów University of Technology, 35-959 Rzeszów, Poland; 139757@stud.prz.edu.pl (K.K.); dorota.stadnicka@prz.edu.pl (D.S.); 2 Faculty of Electrical and Computer Engineering, Rzeszów University of Technology, 35-959 Rzeszów, Poland

**Keywords:** edge computing, intelligent manufacturing, digital factory, Industry 4.0

## Abstract

This article presents the results of research with the main goal of identifying possible applications of edge computing (EC) in industry. This study used the methodology of systematic literature review and text mining analysis. The main findings showed that the primary goal of EC is to reduce the time required to transfer large amounts of data. With the ability to analyze data at the edge, it is possible to obtain immediate feedback and use it in the decision-making process. However, the implementation of EC requires investments not only in infrastructure, but also in the development of employee knowledge related to modern computing methods based on artificial intelligence. As the results of the analyses showed, great importance is also attached to energy consumption, both in ongoing production processes and for the purposes of data transmission and analysis. This paper also highlights problems related to quality management. Based on the analyses, we indicate further research directions for the application of edge computing and associated technologies that are required in the area of intelligent resource scheduling (for flexible production systems and autonomous systems), anomaly detection and resulting decision making, data analysis and transfer, knowledge management (for smart designing), and simulations (for autonomous systems).

## 1. Introduction

Introducing new and improved tools, machines, devices, or techniques into production processes that increase work efficiency or save raw materials or energy leads to technical progress. In the history of industry so far, we can distinguish four breakthrough concepts that have had a huge impact on production systems: Industry 1.0—water and steam mechanization; Industry 2.0—mass production based on electricity; Industry 3.0—increasing production automation based on digitization; and Industry 4.0—digitalization of manufacturing systems. 

Today’s industry is shifting towards a green and digital transformation. There is much emphasis on the implementation of sustainable development. Three aspects of sustainable development have been discussed in the literature: economic, environmental, and social. Industry 4.0 technology can support all of these [1]. Sustainable development is associated with all technologies applied to develop the economy with simultaneous care for the preservation of the natural environment and respect for people [2]. Consequently, there is a constant search for approaches and technologies that can support these activities. For example, the concept of zero defect manufacturing, widely explored in [3], can support all aspects of sustainability. This is because, by preventing defects, we prevent the waste of materials and energy, and, thus, excessive costs and additional work that would have to be completed to eliminate defects or to produce a new product without defects. Therefore, the goal is to prevent defects and achieve correct production the first time. Some technologies are based on the analysis of previously collected data to predict problems, such as virtual metrology, which estimates the results of a process based on previous metrological measurements rather than performing them in real time [4]. In the case of semiconductor manufacturing, a machine learning system is used for this purpose [5]. For this to be possible, it is necessary to collect data for analysis. 

It is indispensable to monitor the processes and ensure that the required parameters of the processes will be maintained throughout the entire production process. For this purpose, it becomes necessary to implement technologies that will enable data collection and analysis in real time, so that, if necessary, parameters can be adjusted immediately. This means that the entire procedure must be carried out at the point of process realization, that is, at the edge. Therefore, to ensure the quality of the process and products (that is, zero defect manufacturing), edge computing (EC) must be implemented [6]. Therefore, it can be said that EC can also support sustainable development. However, for this to be possible, a digital transformation is necessary.

When analyzing the concepts of Industry 4.0, the literature distinguishes the following categories of technologies on which the concept is based: cyber-physical systems (e.g., [7]), Internet of Things, big data analytics, cloud computing, fog and edge computing (EC), Augmented Reality (AR) and Virtual Reality, robotics, cyber security, Semantic Web technologies, and additive manufacturing [8,9].

While browsing the available publications, we notice that some researchers had focused on creating taxonomies for Industry 4.0 technologies that supported the digital transformation and industrial needs [10,11]. Some researchers studied business use cases or consulted company reports and white papers [12]. In [13], the authors, through a systematic literature review, looked for the links between Industry 4.0 and sustainability, while in [14] the authors studied challenges and risks related to Industry 4.0. Additionally, trends and directions for artificial intelligence application in industry [15] and the Industrial Internet of Things [16] have been studied through systematic literature reviews. Similarly, with the use of literature review and multiple case studies, the authors of [17] discussed big data analytics in industrial applications. There are also papers that have widely discussed the use of cloud computing in industry and related security problems [18,19]. However, we did not find any publications presenting literature reviews related to edge computing application in industry. However, for example, in [20] the authors presented a comparison of edge computing architectures found throughout survey-specific areas of application, but edge-computing-related technologies were not summarized.

The main purpose of this study is to identify the possibilities of using edge computing in industry on the basis of literature review. The analysis was conducted to answer the questions of where and how enterprises can apply EC to produce more economically and to respond faster to customer needs, as well as to decrease negative influences on the environment and provide a friendlier workplace for employees. 

This paper consists of five sections. The next section presents the goal and methodology of the study, including research questions, systematic literature review methodology, text mining procedure, and qualitative analysis methodology. The third section presents the research results and the analysis. Then, discussion on the research questions is described. The last section presents conclusions.

## 2. Goal and Methodology of the Research

### 2.1. General Overview and Research Questions

The main goal of this study was to identify the areas of possible EC application in the manufacturing industry. 

The following steps were taken as part of the research:

1. A systematic literature review in the field of EC;
2. Text mining analysis of identified keywords;
3. Qualitative analysis of abstracts and full texts in terms of technologies used in production areas.

The research was conducted to answer the following research questions:

RQ1: What topics are discussed in the literature in relation to edge computing (EC)?

RQ2: What are the possibilities of using EC in the manufacturing industry?

### 2.2. Systematic Literature Review

In this paper, a systematic literature review was applied. A systematic review allows for a critical and reproducible summary of the results on a specific topic [21].

For the research, three databases were searched: Web of Science, IEEE Xplore, and Scopus. The following inclusion criteria were used in the process: (1) language (English), (2) search in (title, abstract, and keywords), and (3) access (works available in full version).

The following combinations of keywords were used in the searching process: “edge computing” AND “manufacturing”, “edge computing” AND “quality control”, “edge computing” AND “machining”, and “edge computing” AND “production”.

The number of publications that appeared in the first search in each database after using a specific combination of keywords is presented in Table 1.

In order to limit the number of publications to those most related to the analyzed issue, the following categories were excluded: agriculture multidisciplinary, biotechnology applied microbiology (Web of Science), agricultural and biological sciences, earth and planetary sciences, environmental science, medicine, multidisciplinary, and social sciences (Scopus). 

**Figure 1:**
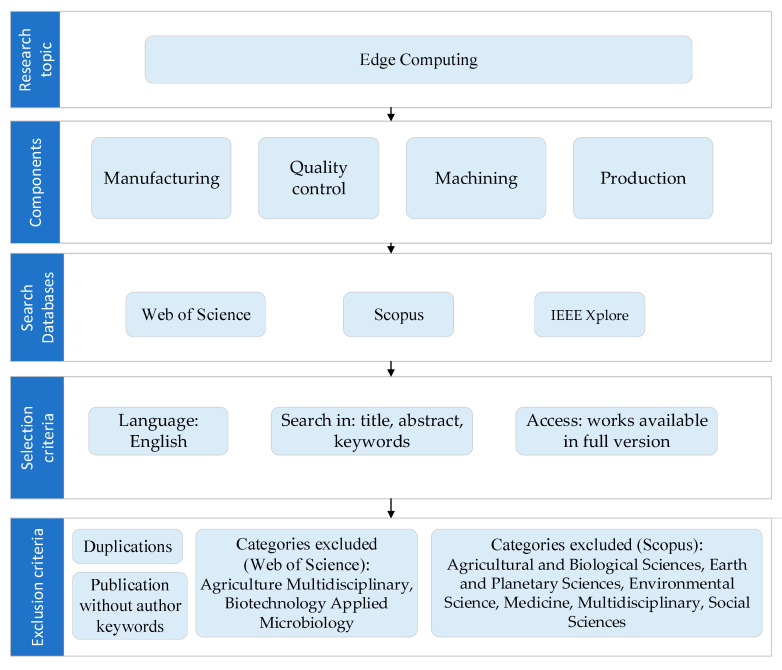
Data extraction plan.

The data extraction plan is presented in Figure 1.

After applying the exclusion criteria, the following results were obtained: 326 publications in Web of Science, 680 publications in IEEE Explore, and 585 publications in Scopus. Then, a joint database was prepared from which duplicates were removed. Thus, 903 publications remained. Then, the publications without keywords were eliminated. Thus, 722 articles were left.

### 2.3. The Text Mining Procedure

Text mining analysis was performed using VOSviewer [22]. A map was created based on the bibliographic data, i.e., keywords, from 722 publications. It required the preparation of one file with data from all the searched databases. Scopus file structure was applied. For the type of analysis and the counting method, author keyword co-occurrence and full counting were selected. In order to identify the most frequently discussed topics, it was assumed that only author keywords that occurred at least 10 times would be taken into consideration. A total of 2039 different author keywords were identified, and 34 of these met the threshold. Table 2 presents a list of the identified author keywords with information about their occurrences and total link strength.

It was noticed that there are words with identical meanings in the table. Therefore, a list of synonyms was developed, and their initial name was indicated (Table 3). Then, the final list of author keywords most often appearing in the analyzed articles was prepared (Table 4). The specified terms were further analyzed. In Section 4.2, we give definitions of these terms. Then, in the next section, the network of terms is visualized.

The most common terms identified through text mining were further analyzed and are presented in the next section.

### 2.4. Qualitative Analysis of Data

For qualitative analysis to limit the number of papers for deep review, from 722 publications, we chose only the papers registered in the Web of Science database. We can consider this a limitation of our study because, in non-reviewed papers, other applications can be presented. However, we decided that adopting this rule would be clear that, when looking for further possible application, it would be advisable to read articles from other databases.

After analyzing the abstracts of publications, the publications related to the manufacturing industry were selected for further analysis. The goal of the analysis was to identify the applications of EC in that industry.

## 3. The Research Results and Analysis

### 3.1. Topic Definitions

The most common terms identified through text mining are defined as follows.

**Edge computing** (EC) is the so-called marginal calculations where data are generated and immediately processed at the edge of the network. This technology is necessary to cope with the growing number of communicating devices connected to the network. The goal is to avoid high latency and bottlenecks in cloud computing traffic in networks where several devices both access and generate large amounts of data. Edge computing also improves network support for mobility, security, and privacy [23].

**Cyber-physical systems** (CPS) are intelligent computer systems that are highly connected, and their physical and computational elements work together [24].

**Internet of Things** is a system that is connected by a network of things, e.g., machines or devices. Due to this, they are able to communicate with each other by processing, collecting, or exchanging data [8].

**Industrial Internet of Things** (IIoT) is the integration of different IoT technologies into industrial manufacturing processes to ensure a high level of efficiency and automation which leads to economic growth. In IIoT, communication technologies are present within the whole manufacturing lifecycle. IIoT is a key element of CPS, supporting the ability to collect data, compute, transfer information, and control processes [25].

**Cloud computing** is a technology for storing and processing data using scalable services on the Internet [8].

**Fog computing** is an extension of the concept of cloud computing that shifts computing to the edge of the network. It is closely related to edge computing and the Internet of Things and has all their advantages. It is the link between edge devices and data centers [26].

**Computation offloading** is the offloading of calculations by moving the location of execution from the cloud to the edge of the network in order to accelerate them and reduce delays [27].

**Blockchain** technology is based on the use of blockchains, which are shared distributer registers. It is used in smart and digitally connected factories to store information about resources and processes. Blockchain technology ensures that the processes are more autonomous, efficient, faster, and secure by providing safe communication mechanisms (a public and private key) to ensure authentication [28].

**Smart factory** enables high personalization of production with little participation from employees. Cyber-physical production systems are constructed in such a way that they can react to almost any change in the market in a short time [29].

**Smart manufacturing** is a new form of production that integrates current and future production assets with sensors, computing platforms, communication technology, control, simulation, modeling, and data-intensive computing with manufacturing engineering [30].

**Artificial Intelligence** (AI) is, according to Andreas Kaplan and Michael Haenlein, “the ability of a system to correctly interpret data from external sources, learn from them and use this knowledge to perform specific tasks and achieve goals through flexible adaptation” [31].

**Machine learning** (ML) is the process of constructing computer programs that are capable of learning from data. Appropriate algorithms allow the software to automate the process of data acquisition and analysis for the purpose of improving and developing its own system [32]. ML can have a positive impact on products and processes by enabling effective prediction of their behavior based on past experience, data, and information [33].

**Deep learning** is an ML category where algorithms (usually artificial neural networks) are composed to form communicating layers. Each layer is taught to transform input into a more meaningful concept (e.g., pixels—edges—a shape—a flower) [34].

**Deep reinforcement learning** is when techniques of deep learning are combined with reinforcement learning methods and used to represent problems related to the introduction of raw multidimensional data [35].

**Mobile edge computing** is a concept that combines elements of information technology and a telecommunications network, in which computing, memory, and network resources are integrated with a cellular base station [36].

**5G** is the fifth generation cellular network standard that is expected to transmit data faster, increase the connectivity spectrum of devices, increase throughput, lower costs, increase consistency, increase quality, and reduce data transmission delays [37].

**Big data** is, according to Jarosław Woźniczka, “diverse and variable data sets created thanks to modern telecommunications devices that are stored, processed and analyzed using advanced information technology” [38].

**Resource allocation** is the allocation of the required resources to the nodes in the network according to the possibility of their use [39].

**Resource management** is the efficient and effective development of an organization’s resources when they are needed [40].

**Game theory** is a mathematical theory of socio-economic phenomena that shows interactions between decision-making units. This theory is based on structural procedures of mathematics and addresses problems from various fields of application [41].

**Digital twin** is a virtual model that is fully compatible and consistent with a physical object. It simulates object behavior and performance in a real-time environment [42]. Digital twins support sustainable development by saving resources, preventing waste, and, thus, optimizing the effort involved [43].

**SDN** (software-defined networking) is a programmable network building technology that enables central management and control. It consolidates all control into one node—a network controller (no distributed control architecture). Network relay devices no longer participate in network control and only forward data packets [44].

**Anomaly detection** uses data mining techniques to detect surprising behaviors hidden in data. When applied to cybersecurity, anomaly detection increases the probability of detecting an attempted break-in or attack [45]. Machine failure can be predicted in machine monitoring with anomaly detection.

### 3.2. Network and Its Visualization

**Figure 2:**
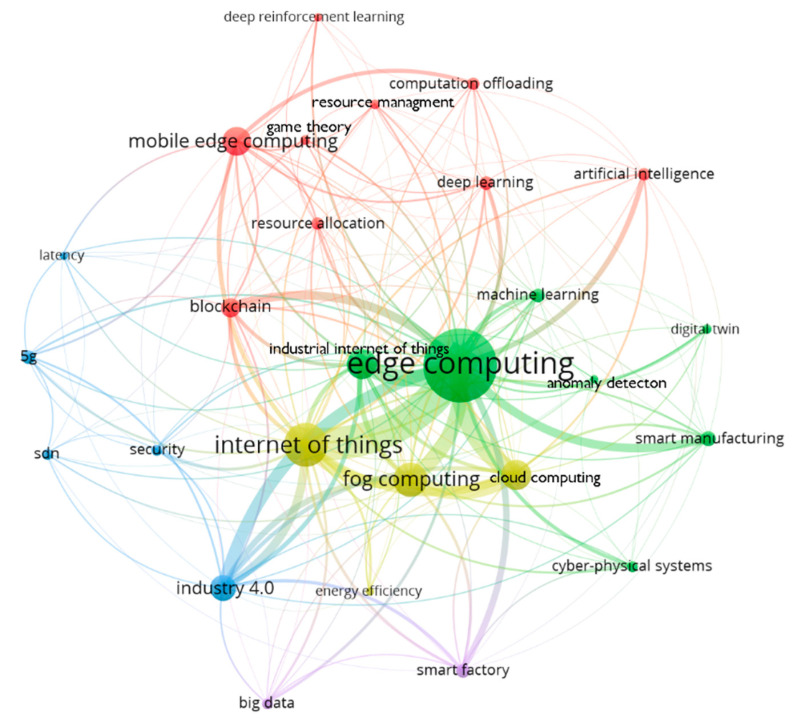
Term network visualization in VOSviewer version 1.6.16.

Based on the mapping of keywords that appeared at least 10 times, the network visualization shown in Figure 2 [46] was obtained after the use of thesaurus grouping. In the visualization, each circle represents a specific term. The area of the circle indicates the number of publications with the appropriate term. The thickness of the line joining the terms indicates the total strength of the term co-occurrences in different works [47].

Terms that often coexisted with each other are placed close to each other in the visualization. The terms were grouped into five clusters. The blue cluster in the left area of the visualization consists of terms related to the decrease in data transfer time. The red cluster in the top area covers AI-related terms. In the right and central visualization areas, the green cluster consists of manufacturing-system-related terms. In the central part of the yellow cluster are terms related to locations where data are computed. At the bottom of the map, the purple cluster contains terms related to data volume. The conducted mapping gives a general view of issues related to EC.

The numerical values of term weights and link strengths are presented in Table 5 and Table 6, respectively. The weights inform about the importance of the terms. Here, it refers to the number of occurrences of a term in publications. The link strength provides information about the degree of association between the term *Edge Computing* and a term shown in the first column of Table 6. The absolute value is the number of publications in which both terms occurred in the keywords section. Relative values were calculated as a percent of the maximum value from the given category.

In Table 5, the absolute values of the weights do not sum up in a table row. For example, the number of occurrences of the term *Edge Computing* in all three databases was 346 and 346 ≠ 138 + 180 + 218. The reasons for this phenomenon are as follows:

1. The same publication could have been indexed in multiple databases;
2. The number of occurrence of a term in a database was less than 10 (in such cases, the term was not included in the results).

In Table 6, the sum of the absolute values in the column *link strength* for all databases was 397, which was the total link strength for the term *Edge Computing* (see Table 4, row 1). However, as in Table 5, the absolute value of *link strength* for a selected term does not sum up. The reasons are identical to those mentioned earlier.

### 3.3. Identified Challenges and Technologies Related to EC in Production Systems

An analysis of the full articles retrieved from the WoS database allowed for the identification of EC industrial applications and technologies related to the EC. The results of the analysis are summarized in Table 7. 

Apart from edge computing, the following technologies were indicated in the analyzed works: 5G, blockchain, AR, Mixed Reality, HoloLens, discrete-event simulation, big data, CPS, data analytics, data mining, cloud computing, fog computing, fog-edge computing, AI, ML, deep learning, reinforcement learning, deep reinforcement learning, inverse reinforcement learning, neural networks, deep neural network, convolutional neural networks, distributed ensemble learning, dynamic knowledge bases, emotion interaction, facial recognition, image mining, mobile cloud computing, mobile edge computing, mobile edge-cloud computing, particle swarm optimization, evolutionary algorithm, programmable computer network (SDN), and programmable gate arrays.

## 4. Discussion

### 4.1. Topics Related to Edge Computing

**Figure 3:**
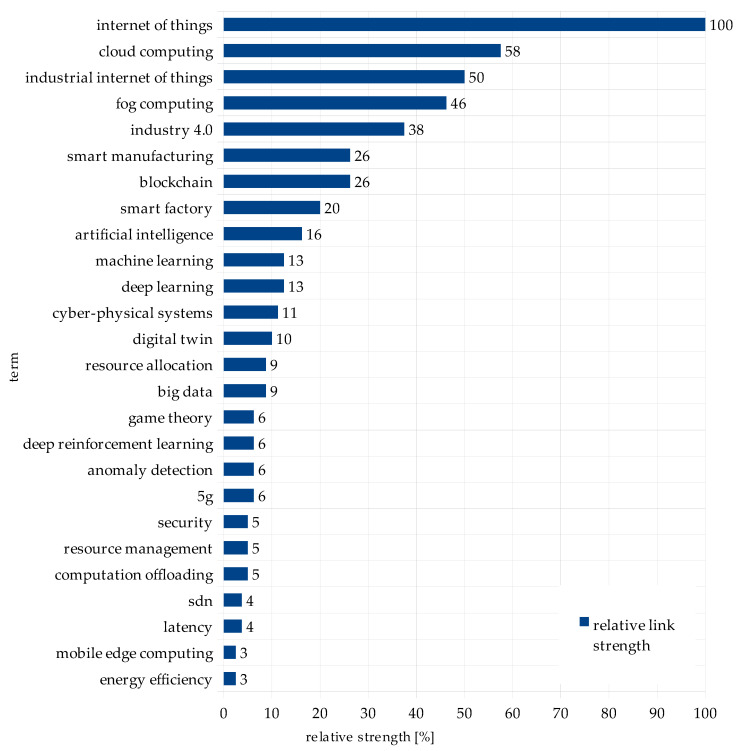
Relative link strength between the term *Edge Computing* and selected terms.

The aim of this study was to discover the possibility of using edge computing in industry based on the existing scientific evidence. The first research question (RQ1) asked about the topics discussed in the literature in relation to edge computing. The answer was that the most-discussed topics were connected with the Internet of Things, cloud computing, Industrial Internet of Things, and fog computing. The topics that were least discussed in connection with EC were energy efficiency, mobile edge computing, latency, and SDN. These conclusions were drawn from the analysis of Table 6 and Figure 3.

Based on relative link strength (RLS) values from Table 6, we evaluated content differences between individual bibliographic databases. Table 8 presents a proposal of such an evaluation calculated as an arithmetic difference (the RLS of a term for a selected database minus the RLS of a term for the whole set) (Equation (1)):

Δ = RLS_SINGLE_DB_ − RLS_ALL_. (1)

where RLS_SINGLE_DB_ is the relative link strength for a single database and RLS_ALL_ is the relative link strength for all databases.

Values of Δ provide information about the extent to which the content of publications in a single database differs from the whole set. Negative values of Δ indicate that there are fewer related terms in the given database compared to the whole. In order to obtain an aggregate coefficient of database differences, we proposed calculation of the root mean square (RMS) for the Δ. The calculated values of RMS_Δ_ were: RMS_ΔWOS_ = 5.3, RMS_ΔIEEE_Ex_ = 4.9, and RMS_ΔSCOPUS_ = 4.3.

Taking into account the obtained numerical values, the following conclusions can be proposed:

1. In each of the three databases, the most- and the least-discussed topics related to the *Edge Computing* term were the same as mentioned previously (see Table 6, last four rows);
2. Compared to the dataset composed of WoS, IEEE Xplore, and Scopus, WoS contained fewer papers in which the term *Edge Computing* was connected to the terms *Cloud Computing*, *Industry 4.0*, *Artificial Intelligence*, and *Resource Allocation*;
3. The IEEE Xplore database had more publications where the term *Edge Computing* was connected to the term *Industrial Internet of Things* and fewer connected to the term *Digital Twin*;
4. The SCOPUS database contained fewer papers in which the term *Edge Computing* was connected to the term *Industrial Internet of Things*;
5. The overall content of the considered databases in the context of terms related to EC was similar. Therefore, for a more-detailed publication analysis, one database can be chosen instead of the whole set. In such a case, we expect that analysis results will have an error within the limits of RMS_Δ_.

### 4.2. Edge Computing Possible Applications

The second research question (RQ2) inquired about the possibilities of using edge computing in the manufacturing industry. These possibilities were discovered by analyzing problems discussed in the reviewed publications. After reviewing 119 papers, 32 main challenges were identified, as presented in Table 7 (Section 3.3). Based on the information included in Table 7, we proposed to distinguish the following 12 main groups based on the area of occurrence. 

Group 1: intelligent manufacturing organization in a form of **CPS** that includes process and data transfer automation, as well as data analytics realized in the cloud and on the edge. The aim is to improve the efficiency of production processes by remote control and optimization of response time. Moreover, distributed AI supports edge networks, which may extend to a supply chain.

Group 2: **data management** and data security covering data acquisition and transfer, as well as cyber attack prevention. The goal is to collect data and transfer it securely to the destination, while ensuring minimum energy consumption. This requires, among others, implementing appropriate security protocols, identifying threats, and preventing cyber attacks.

Group 3: **real-time** data processing to monitor the manufacturing process, machines, the quality of the manufactured products, or product performance. In the reviewed papers, different examples were presented, such as monitoring of the work of aircraft engines and their components or production machines and different characteristics. The aim is to evaluate the performance, evaluate the condition, or evaluate the life remaining.

Group 4: **quality** control supported by technologies. The goal is to shorten and automate quality control processes using historical data, as in the case of virtual metrology systems [3]. Visual inspection and image recognition are also of great importance in this group as detection of anomalies in production can be realized in real time.

Group 5: the use of **simulation** to improve manufacturing processes. Examples presented in the literature concerned, among others, digital twins that can be used in simulations or in real production monitoring and adjustment. 

Group 6: improvement of **communication** and interactions between machines, as well as between machines and humans. This group is mainly about cooperation remaining undisturbed by communication breaks or erroneous messages that can cause errors, cause delays, and generate costs.

Group 7: facilitating **IT system** development. The main goal is the development of IT solutions based on data and tailored to needs as much as possible.

Group 8: **knowledge management**. The goal is to provide relevant knowledge wherever it is needed. An additional advantage is the automatic generation of knowledge based on data.

Group 9: supporting the **designing** process. In the presented examples, AI was applied to support designing processes in the field of decision making.

Group 10: **energy consumption** and saving in manufacturing and data management processes. The presented challenges were related to energy savings not only in production, but also in data processing. The necessity to collect large amounts of data increases the energy consumption of devices for recording, analyzing, and transferring data.

Group 11: manufacturing process **scheduling** and resource allocation. The goal is to involve AI in optimizing the use of resources and planning their involvement in the implementation of production processes with ongoing monitoring of demand and immediate response to it. This is connected with lean manufacturing, Just-in-Time, and pull system concepts, which allow the minimization of resource consumption.

Group 12: **robots** and **autonomous systems**. In autonomous and robotic systems, many decisions are made depending on the situation. An unmanned system must react appropriately to the situation in order to achieve the set goal.

Based on the research results, it can be said that EC can be applied in different domains. EC is primarily used because of its ability to collect big data and the increased difficulties with fast data transfer. Although 5G technology is more and more widely used, by implementing EC, it was discovered that it was no longer necessary to transfer all data to the central system, since it can be used on the edge. This can, for example, reduce energy consumption, as well as the space needed for data collection. At the edge, the data can be used in real time, and only those that are unique in some way can be collected and transferred to a central system.

### 4.3. Identified Directions for Further Development and Research in the Areas of Edge Computing Application

**Figure 4:**
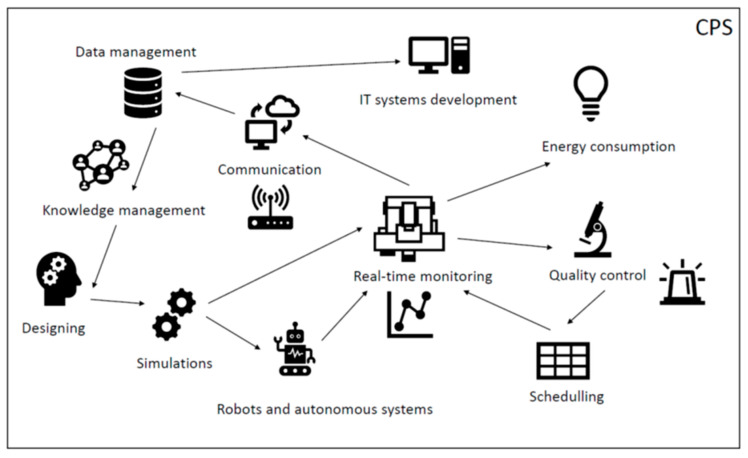
EC in CPS applications summary.

The identified challenges (which are described in the previous section) in which EC was applied are summarized in Figure 4. The identified applications of EC are related to CPS. The processes realized in CPS must be scheduled. Therefore, an intelligent resource scheduling strategy can be applied to fulfill the real-time requirement needing to be taken into consideration in smart manufacturing supported by edge computing [145]. The required adjustments to the production schedule may result from changes in customer demand, as well as the quality of the production. This means, for example, that the production of a non-conforming product will generate an automatic schedule change to ensure that a certain number of good products are produced for customers. This is an important direction of development because the introduction of such solutions will eliminate overproduction resulting from the production of more products caused by production planning based on statistics from historical data on the percentage of non-conforming products produced. This is related to the detection of production errors in real time, the identification and classification of defects, and a decision support system in product quality control. Therefore, the development of methods and tools facilitating product control during the production process is important to detect non-conformities [106] at the location of occurrence and not to transfer non-conforming products to the next stage of the production process, which only generates costs and other problems.

Not only should the manufactured products be controlled in real time, but most of the implemented processes and devices must be monitored to estimate the possibility of problems [77]. An important direction of further development in this area is the implementation of decision-making systems at the edge to prevent the production of a non-conforming product. Certain ranges of process parameter values (e.g., speed and rotation) or the occurrence of specific values of the operating parameters for machines and devices (e.g., power consumption and temperature) may result in the appearance of a non-compliant product. Therefore, the ability to identify such situations in order to react quickly to them is something desirable to implement in this industry.

The data coming from the monitoring process can be transferred through communication systems to a database to be stored. A “hold-until-changed” approach can be applied to decide which data should be stored. This approach is based on keeping track of earlier transmitted data to determine which data should be transmitted when and to whom [130]. This can minimize data storage requirements and associated costs and result in lower bandwidth utilization, as well as high-speed transmission. It is an important research direction related to sustainable development. In addition, it can reduce the amount of work involved in further data analysis, the results of which will not add any value to the decision-making process.

One of the topics that appears in the literature in an EC context is energy consumption. This can be related to data transfer in CPS [101] and production scheduling considering energy consumption [150]. The number of connected devices in the CPS has increased significantly, which has generated an energy demand. Therefore, we need research and innovative solutions that will not require much energy.

The data coming from the monitoring process can be transferred through communication systems to a database where they can be transformed into knowledge available for employees. Knowledge management is important from many perspectives, for example, in the management of product lifecycle data and in supporting related decision-making processes [66]. Therefore, products can be better-suited to changing requirements. Agile adaptation to market needs and flexible production systems are the directions in which enterprises should develop. Modern technologies should support enterprises looking in this direction.

Another identified important direction of research and subsequent industrial implementations is simulations. Although many simulation methods and tools are currently known, their use remains time-consuming and, thus, expensive. The newly designed processes and product operations can be simulated before implementation, as well as after implementation, for example, with the use of digital twins [43]. This is especially important for flexible production systems, as well as for autonomous systems [157]. In this context, the direction of further research that we have identified is the development of methods and tools facilitating the creation of models of the functioning of real objects and simulating their work in various conditions in order to identify potential problems that may arise during operation.

## 5. Conclusions

With the use of a systematic review of the literature, it was possible to analyze widely the use of EC. Applied text mining analysis enabled automated text searching of a vast number of publications in order to obtain a new type of information, constituting a quantitative data analysis. A qualitative analysis of the abstracts and full papers allowed the identification of documented application areas of edge computing in industry. The results of the analyses indicated both the technologies used to analyze the identified problems and the documented sources of data.

The presented results are useful material for enterprises looking for answers to questions focusing on smart manufacturing implementation and decision makers in manufacturing companies to determine the possibility of implementing edge computing and related technologies.

The main conclusion of this study was that, despite the large number of publications on edge computing, this field of knowledge is still being developed. It is a promising field of scientific exploration for the future, and many research problems still need to be solved. 

Future research in EC should particularly cover areas where actions require immediate decision making and the processing of big data, such as autonomous control of robots, vehicles, or entire factories. The second area of research should be technologies related to mobile edge computing, for example, telecommunications and all issues related to 5G technology.

In particular, on the basis of the conducted research, we have indicated areas that require further research due to the need to implement innovative solutions in the industry. The further research on the application of edge computing and associated technologies is required in the area of intelligent resource scheduling (especially for flexible production systems and autonomous systems), anomaly detection and the resulting decision making (e.g., improper operation of machines, improper implementation of processes, and appearance of non-conforming products), data analysis and transfer (When? How? What? To whom?), knowledge management (knowledge used for smart designing), and simulations (to prevent future problems, especially in autonomous systems).

Further literature research may help identify other directions for the development of EC applications. But the development of effective and practically applicable solutions in the areas indicated by this paper that enterprises will be able to implement would be a significant step towards the development of smart factories.

The systematic literature review also applied to the taxonomy building process. Examples are papers [167] or [168], where the authors presented proposals for the taxonomy of EC in intelligent manufacturing and a general taxonomy for the EC paradigm. The first of the taxonomies contained a section with potential applications of EC in the industry; however, it was limited to a few examples. The second paper did not mention applications of EC in manufacturing. The results of the work presented in this paper can be used to enrich available taxonomies or to create new taxonomies in the area of EC application in the manufacturing industry.

## Figures and Tables

**Table 1:** Number of results displayed when searching electronic databases.

Keywords Combination	Web of Science	IEEE Xplore	Scopus	Total
“edge computing” AND “manufacturing”	146	259	254	659
“edge computing” AND “production”	162	328	281	771
“edge computing” AND “quality control”	6	8	90	104
“edge computing” AND “machining”	13	85	16	114

**Table 2:** An initial list of terms with a threshold of 10 occurrences.

Keyword	Occurrences	Total Link Strength	Keyword	Occurrences	Total Link Strength
edge computing	346	397	resource allocation	18	33
fog computing	97	186	big data	14	31
cloud computing	76	166	computation offloading	18	29
Internet of Things	75	146	cyber-physical systems	15	28
Industry 4.0	60	129	Industrial IoT	10	27
IoT	47	91	security	14	27
blockchain	36	72	Industrial Internet of Things (IIoT)	22	26
smart factory	23	56	resource management	12	26
smart manufacturing	24	52	game theory	12	25
Internet of Things (IoT)	31	50	digital twin	12	21
machine learning	21	45	energy efficiency	11	21
IIoT	16	43	SDN	14	21
mobile edge computing	37	41	latency	10	20
deep learning	23	40	anomaly detection	11	16
Industrial Internet of Things	25	40	deep reinforcement learning	11	15
5G	23	35	mec	16	14
artificial intelligence	20	35	mobile edge computing (mec)	17	10

**Table 3:** The list of synonyms and their valid names.

Synonyms	Valid Name	Synonyms	Valid Name
Industrial Internet of Things (IIoT) Industrial Internet of Things; IIot Industrial IoT Industrial IoT (IIoT) Industrial Internet of Things (IIoTs) Industry IoT	Industrial Internet of Things	Internet of Things Internet of Things (IoT); IoT Internet of Things (IoT)	Internet of Things
		Mobile edge computing; MEC Mobile edge computing (MEC) Mobile-edge computing (MEC)	Mobile edge computing

**Table 4:** The final list of terms.

Keyword	Occurrences	Total Link Strength	Keyword	Occurrences	Total Link Strength
edge computing	346	397	resource allocation	18	33
Internet of Things	144	258	big data	14	30
fog computing	97	180	computation offloading	18	30
cloud computing	76	163	cyber-physical systems	15	28
Industrial Internet of Things	78	139	security	14	28
Industry 4.0	60	127	resource management	12	27
blockchain	36	74	game theory	12	26
mobile edge computing	73	74	digital twin	12	21
smart factory	23	56	latency	10	21
smart manufacturing	24	52	SDN	14	20
machine learning	21	44	energy efficiency	11	21
deep learning	23	40	anomaly detection	11	16
artificial intelligence	20	35	deep reinforcement learning	11	15
5G	23	34			

**Table 5:** Term weights in the analyzed databases.

Term	All Databases		WoS		IEEE Explorer		SCOPUS	
	Weight	Relative Weight	Weight	Relative Weight	Weight	Relative Weight	Weight	Relative Weight
latency	10	2.9	0	0.0	0	0.0	0	0.0
anomaly detection	11	3.2	6	4.3	0	0.0	0	0.0
deep reinforcement learning	11	3.2	0	0.0	7	3.9	0	0.0
energy efficiency	11	3.2	0	0.0	8	4.4	0	0.0
digital twin	12	3.5	7	5.1	0	0.0	9	4.1
game theory	12	3.5	0	0.0	9	5.0	0	0.0
resource management	12	3.5	0	0.0	9	5.0	0	0.0
big data	14	4.0	4	2.9	10	5.6	7	3.2
SDN	14	4.0	0	0.0	8	4.4	6	2.8
security	14	4.0	0	0.0	11	6.1	0	0.0
cyber-physical systems	15	4.3	5	3.6	7	3.9	10	4.6
computation offloading	18	5.2	0	0.0	15	8.3	6	2.8
resource allocation	18	5.2	0	0.0	14	7.8	6	2.8
artificial intelligence	20	5.8	5	3.6	9	5.0	14	6.4
machine learning	21	6.1	11	8.0	14	7.8	18	8.3
5G	23	6.6	7	5.1	10	5.6	14	6.4
deep learning	23	6.6	8	5.8	14	7.8	12	5.5
smart factory	23	6.6	11	8.0	13	7.2	13	6.0
smart manufacturing	24	6.9	13	9.4	13	7.2	19	8.7
blockchain	36	10.4	10	7.2	23	12.8	15	6.9
Industry 4.0	60	17.3	17	12.3	38	21.1	33	15.1
mobile edge computing	73	21.1	12	8.7	50	27.8	31	14.2
cloud computing	76	22.0	20	14.5	45	25.0	38	17.4
Industrial Internet of Things	78	22.5	26	18.8	57	31.7	34	15.6
fog computing	97	28.0	25	18.1	70	38.9	37	17.0
Internet of Things	144	41.6	47	34.1	79	43.9	91	41.7
edge computing	346	100.0	138	100.0	180	100.0	218	100.0

**Table 6:** Link strength between a term and the *Edge Computing* term.

Term	All Databases		WoS		IEEE Explorer		SCOPUS	
	Link Strength	Relative Link Strength	Link Strength	Relative Link Strength	Link Strength	Relative Link Strength	Link Strength	Relative Link Strength
energy efficiency	2	2.5	0	0.0	0	0.0	0	0.0
mobile edge computing	2	2.5	1	2.6	1	2.6	1	1.8
latency	3	3.8	0	0.0	0	0.0	0	0.0
SDN	3	3.8	0	0.0	3	7.9	1	1.8
computation offloading	4	5.0	0	0.0	3	7.9	3	5.4
resource management	4	5.0	0	0.0	2	5.3	0	0.0
security	4	5.0	0	0.0	3	7.9	0	0.0
5G	5	6.3	3	7.9	2	5.3	3	5.4
anomaly detection	5	6.3	1	2.6	0	0.0	0	0.0
deep reinforcement learning	5	6.3	0	0.0	2	5.3	0	0.0
game theory	5	6.3	0	0.0	4	10.5	0	0.0
big data	7	8.8	3	7.9	5	13.2	5	8.9
resource allocation	7	8.8	0	0.0	4	10.5	4	7.1
digital twin	8	10.0	4	10.5	0	0.0	7	12.5
cyber-physical systems	9	11.3	3	7.9	4	10.5	5	8.9
deep learning	10	12.5	3	7.9	6	15.8	5	8.9
machine learning	10	12.5	5	13.2	6	15.8	9	16.1
artificial intelligence	13	16.3	3	7.9	6	15.8	9	16.1
smart factory	16	20.0	9	23.7	8	21.1	9	16.1
blockchain	21	26.3	8	21.1	12	31.6	12	21.4
smart manufacturing	21	26.3	12	31.6	11	28.9	18	32.1
Industry 4.0	30	37.5	10	26.3	16	42.1	18	32.1
fog computing	37	46.3	17	44.7	18	47.4	23	41.1
Industrial Internet of Things	40	50.0	19	50.0	25	65.8	22	39.3
cloud computing	46	57.5	17	44.7	19	50.0	31	55.4
Internet of Things	80	100.0	38	100.0	38	100.0	56	100.0

**Table 7:** EC industrial application and related technologies.

Industrial Application	Source	Technologies
Machine-to-machine communication	[48,49]	Cloud computing, discrete-event simulation
Human–machine interaction	[50]	CPS
Front-end IoT devices	[51]	Fog computing
Robot calibration, dynamic reorganization, and reconfiguration of the assembly line	[52,53]	Deep learning
Creation of a digital twin, adaptive production, Digital Shadow	[54,55,56,57,58,59]	Data mining, dynamic knowledge bases, cloud and fog computing
CNC machining machine simulation	[60]	AR, CPS, HoloLens
Discovery of data-driven solutions, efficiency and flexibility of IT systems, IT system development	[61,62]	Mixed Reality
Sharing knowledge and services in production ecosystems	[63]	Blockchain
Improvement of the efficiency of production process	[64,65]	ML, AI, emotion interaction
Product design evaluation, AM-based product development process	[66,67]	Cloud computing, AI
Product damage diagnostics, diagnostics and prognostics in industrial applications	[68,69]	Deep learning, distributed ensemble learning
Diagnostics of machine part damage	[70,71]	Deep neural network,
Assessment of the condition of working aircraft engines and predicting remaining service life of components	[72]	deep learning
Monitoring and damping of spindle vibration	[73]	Cloud computing
Reduction of energy consumption, planning of energy resources, minimizing delays and power consumption	[64,74,75]	AI, Mobile edge computing, particle swarm optimization
Real-time data processing, real-time industrial automation monitoring, real-time surface roughness monitoring, monitoring and damping of spindle vibration, analysis of the thermal characteristics of machine tool spindles	[73,76,77,78,79,80,81]	CPS, cloud computing, 5G, programmable computer network (SDN)
Intelligent manufacturing, production automation, CPS, increasing efficiency, automation, remote operation and monitoring, remotely controlled manufacturing, edge-cloud cooperation, optimizing response time of microservice-based applications, intelligent and flexible manufacturing	[82,83,84,85,86,87,88,89,90,91,92,93,94,95,96,97,98,99,100,101,102]	Cloud computing, data analytics, blockchain, CPS, AI, deep learning, reinforcement learning, particle swarm optimization, mobile edge computing, fog computing, SDN
Visual inspection of products, image edge detection and defect detection, identification and classification of defects, decision support system for product quality control, virtual metrology system, reducing inspection cycle time, quality assurance	[103,104,105,106,107,108,109,110]	ML, cloud computing, convolutional neural network, fog computing, deep learning, image mining
Visual system for product sorting	[111]	Convolutional neural networks, cloud computing
Data acquisition and management, data transfer, data control automation, and security improvement	[112,113,114,115,116,117,118,119]	Fog computing, ML, mobile edge computing, 5G, deep and inverse reinforcement learning
Improving data security, real-time security monitoring, shortening data processing time, reducing energy consumption, anomaly detection, intelligent networks, cyber attack prevention	[120,121,122,123,124,125]	Programmable gate array, AI, big data, cloud computing, ML, deep learning
Energy consumption of uploading data, computing energy waste, efficient data processing, big data real-time feedback, real-world datasets, industrial network, industrial wireless network	[126,127,128,129,130,131,132,133,134,135]	Reinforcement learning, mobile edge-cloud computing, SDN, mobile edge computing
Allocation of resources and machines	[136]	Evolutionary algorithm
Recognition of facial expressions, image restoration	[137,138]	Facial recognition, deep learning
Discovery of edge networks, distributed AI as a service, self-configuration of the network	[139,140,141]	AI, fog computing, 5G, SDN, cloud computing
Detection of production anomalies, anomaly detection in time series data for edge computing, real-time fault detection	[142,143,144]	Convolutional neural networks, neural networks, deep learning
Online job scheduling for networks, infrastructure, dynamic and green scheduling, planning and scheduling of process and resources allocation and utilization, real-time scheduling, task sorting by priority and decision making customized production, cloud MES	[145,146,147,148,149,150,151,152,153,154,155,156]	Neural networks, fog computing, AI, mobile cloud computing, reinforcement learning, cloud computing
Collaboration of heterogeneous robots, autonomous vehicle, and autonomous mobile robots, machine–cloud communication, autonomous navigation	[157,158,159,160,161,162,163,164]	Cloud, mobile edge and fog-edge computing
Flexible distributed networked production, streamlining supply chain management	[165,166]	Cloud computing

**Table 8:** Differences in the relative link strength between all data and individual databases.

Term	Relative Link Strength Differences		
	Δ WOS	Δ IEEE Xplore	Δ SCOPUS
energy efficiency	−2.5	−2.5	−2.5
mobile edge computing	0.1	0.1	−0.7
latency	−3.8	−3.8	−3.8
SDN	−3.8	4.1	−2.0
computation offloading	−5.0	2.9	0.4
resource management	−5.0	0.3	−5.0
security	−5.0	2.9	−5.0
5G	1.6	−1.0	−0.9
anomaly detection	−3.6	−6.3	−6.3
deep reinforcement learning	−6.3	−1.0	−6.3
game theory	−6.3	4.3	−6.3
big data	−0.9	4.4	0.2
resource allocation	−8.8	1.8	−1.6
digital twin	0.5	−10.0	2.5
cyber-physical systems	−3.4	−0.7	−2.3
deep learning	−4.6	3.3	−3.6
machine learning	0.7	3.3	3.6
artificial intelligence	−8.4	−0.5	−0.2
smart factory	3.7	1.1	−3.9
blockchain	−5.2	5.3	−4.8
smart manufacturing	5.3	2.7	5.9
Industry 4.0	−11.2	4.6	−5.4
fog computing	−1.5	1.1	−5.2
Industrial Internet of Things	0.0	15.8	−10.7
cloud computing	−12.8	−7.5	−2.1
Internet of Things	0.0	0.0	0.0

## Data Availability

Not applicable.
